# Synthesis of an Alternated Heterobimetallic Supramolecular Polymer Based on Ru(II) and Fe(II)

**DOI:** 10.3390/molecules25225261

**Published:** 2020-11-11

**Authors:** Manas Kumar Bera, Yoshikazu Ninomiya, Masayoshi Higuchi

**Affiliations:** Electronic Functional Macromolecules Group, Research Center for Functional Materials, National Institute for Materials Science (NIMS), 1-1 Namiki, Tsukuba, Ibaraki 305-0044, Japan; BERA.ManasKumar@nims.go.jp (M.K.B.); NINOMIYA.Yoshikazu@nims.go.jp (Y.N.)

**Keywords:** heterometallic supramolecular polymer, bimetallic, terpyridine, iron, ruthenium

## Abstract

A heterobimetallic supramolecular polymer (polyRuFe) with alternately complexed Ru(II) and Fe(II) is prepared following a stepwise synthetic route through harnessing first the strongly binding metal ion Ru(II) and then the weakly binding metal ion Fe(II). A high yield of product is achieved in each step. The heterometal ions are incorporated into the polymer chain in identical coordination environments formed by two 2,2′:6′,2″-terpyridine moieties. Characterization is accomplished by NMR spectroscopy, MALDI–TOF mass spectrometry, UV–Vis spectroscopy, and cyclic voltammetry. PolyRuFe shows a wide optical window (*λ* = 311–577 nm) and a broad distinct reversible redox nature of two types, originated from the coupling of the two heterometallic segments into the polymer chain. Such characteristics of polyRuFe suggest its potential for various electrochemical and electro-optical applications.

## 1. Introduction

Over the last 40 years, supramolecular chemistry has drawn a large research interest, which brought the Nobel Prize in Chemistry in 1987 [1]. Supramolecular chemistry involves various noncovalent interactions such as coordinate covalent bonding, hydrogen bonding, π–π interactions, and van der Waals interactions, which are generally employed to construct supramolecules and supramolecular polymers. Supramolecular polymers are polymers of repeating units held together by non-covalent interactions. The inclusion of metal ions into a supramolecular polymer, results in a metallo-supramolecular polymer (MSP) [2]. MSPs are generally synthesized by coordination-driven self-assembly (1:1 complexation) of ditopic organic ligands and metal ions [3,4,5]. Therefore, the hybridization of organic and inorganic moieties in MSPs produces unique features, which allow MSPs to be used in a wide range of applications, such as memory devices, molecular motors, sensors, photovoltaic devices, electrochromic devices, light-emitting diodes, proton conduction, and stimuli-responsive materials [3,6,7,8,9].

Although various structural MSPs (linear and branched) have been developed to date, MSPs with a linear structure draw more attention for various applications due to their solution processability. The most common organic ligand that is used to prepare MSPs is te 2,2′:6′,2″-terpyridine (tpy) [10]. This tpy shows high binding affinity for metal ions through tpy–M^2+^–tpy connectivity [11]. To date, various metal ions such as Fe, Ru, Os, Ni, Co, Cu, Zn, and Cd have been used to create monometallic MSPs with a linear structure [4,12]. MSPs containing two heterometal ions have drawn much attention in recent years because of their attractive functionalities which are mainly originated from the coupling of heterometallic segments into the MSP chain. For example, Stang and co-workers synthesized a Pt(II)–Zn(II)-based heterometallic supramolecular polymer with self-healing properties [13]. Our group reported a heterobimetallic MSP containing Fe(II)–Os(II) with unique electro-optical properties [14,15]. The synthesis of heterobimetallic supramolecular polymers is generally achieved by two strategies. One strategy is the introduction of two heterometal ions into the polymer chain in different coordination environments, which is achieved through different binding affinity of the ligand for different metal ions [16]. Another strategy is the introduction of heterometal ions into the MSP chain in identical coordination environments, which is synthetically challenging [14]. In this respect, it should be mentioned that heterobimetallic supramolecular polymers having heterometal ions in identical coordination environments, are of particular interest because the properties of the two heterometallic segments are comparable. If the coordination environments of the heterometal ions in the MSP are different, we cannot compare the properties of individual metal complexes, because the electronic environment of the coordinated metal ions is influenced by the nature of the coordinating ligand.

To date, heterobimetallic supramolecular polymers containing heterometal ions in identical coordination environments are limited to only Os(II) and Fe(II) ions. Other combinations of heterometal ions could enable different optical and electrochemical properties and hence could result in new features. On this basis, herein, we extend the use of metal ions and report the synthesis of a new heterobimetallic supramolecular polymer (polyRuFe) with alternate introduction of Ru (II) and Fe(II) ions, with Ru(II) and Fe(II) ions situated in identical coordination environments made by two tpy units.

## 2. Results and Discussion

**Scheme 1:**
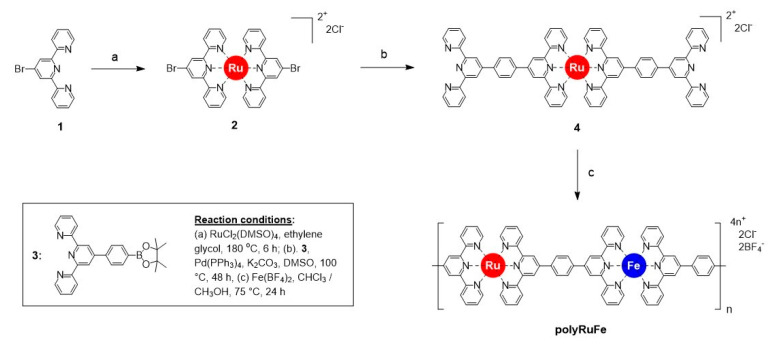
Stepwise synthetic route of a metallic supramolecular polymer containing Ru(II) and Fe(II) ions (polyRuFe).

The synthesis of a heterobimetallic supramolecular polymer (polyRuFe) containing Ru(II) and Fe(II) ions in alternate fashion, was done in a stepwise manner by harnessing first the strong coordination metal ion Ru(II) and then the weak coordination metal ion Fe(II). In this context, it should be mentioned that an approach to introduce Ru(II) and Fe(II) alternately into an MSP chain was done previously, but the synthetic route was not so specific to lead to alternate positions of the two heterometal ions [17]. Here, we overcome that problem through stepwise complexation of Ru(II) and Fe(II) ions, confirming the alternate position of two heterometal ions into the polymer chain. PolyRuFe was synthesized in three steps, as shown in Scheme 1. Using 4′-bromo-2,2′:6′,2′′-terpyridine (compound **1**) as a coordinating ligand and RuCl_2_(DMSO)_2_ as a salt, a Ru(II)-complex (compound **2**) was prepared with 91% yield by refluxing the ligand and salt in ethylene glycol. Then, Suzuki coupling of compound **2** with compound **3** in DMSO at 100 °C in the presence of Pd(PPh_3_)_4_ as a catalyst produced a modified ditopic ligand (compound **4**) with 76% yield. Characterization of all compounds was done by NMR and MALDI–TOF mass spectroscopy (see Materials and Method section for details of the synthetic procedures and Supplementary Materials for NMR and mass spectra (Figures S1–S6)).

**Figure 1:**
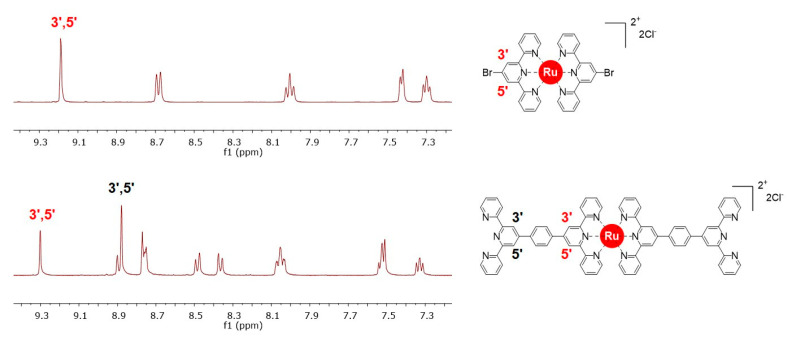
Comparison of the partial ^1^H NMR spectra of compound **2** and compound **4** in CD_2_Cl_2_/CD_3_OD (1:1, *v*/*v*); different types of 3′,5′ peaks are indicated.

As shown in Scheme 1, compound **4** can be considered as a ditopic ligand that contains Ru(II) complexed with two free 2,2′:6′,2′′-terpyridine (tpy) units in opposite direction, which allows for further complexation with another metal ion like Fe(II). In compound **4**, two types of tpy units are present: one type consists of coordinated tpy units, and the other of noncoordinated tpy units. A comparison of the partial ^1^H NMR spectra of compound **2** and compound **4** is shown in Figure 1, which clearly indicates the presence of two types of tpy units in compound **4**. The NMR spectrum of compound **2** showed 3′,5′ resonances of coordinated tpy units at δ = 9.19, whereas compound **4** exhibited two different 3′,5′ resonances at δ = 9.30 and 8.90 ppm for coordinated and noncoordinated (free) tpy unites, respectively.

**Figure 2:**
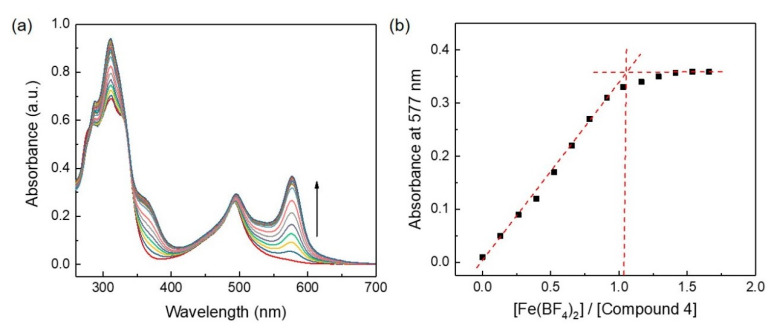
(**a**) Change in the UV–vis absorption of compound **4** (5 × 10^−6^ M in DCM/MeOH 1:1, *v*/*v*) upon gradual addition of Fe(BF_4_)_2_ in MeOH. (**b**) Plot of the absorption of compound **4** at 577 nm as a function of the [Fe(BF_4_)_2_]/[compound **4**] ratio.

The complexation of compound **4** (5 × 10^−6^ M in DCM/MeOH 1:1, *v*/*v*) with Fe(II) ions [like Fe(BF_4_)_2_ 6H_2_O salt in MeOH] was studied by monitoring UV–vis absorption (Figure 2). Compound **4** showed two absorption bands (λ) at 311 and 492 nm. The absorption band at 311 nm corresponded to π–π* transition, and the absorption band at 492 corresponded to metal-to-ligand charge-transfer (MLCT) absorption of the Ru(II)–terpyridine complex. Upon gradual addition of the solution of Fe(II) ion to the solution of compound **4**, an additional absorption band appeared at 577 nm (Figure 2a). This new absorption band at 577 nm corresponded to the MLCT absorption band of the Fe(II) complex, which formed upon complexation of Fe(II) ions with free terpyridine units of compound **4**. The intensity of the MLCT band at 577 nm gradually increased upon increasing the concentration of Fe(II) and achieved saturation when the molar ratio of [Fe(BF_4_)_2_] to [compound **4**] was 1:1 (Figure 2b). Additionally, a small red shift (~3 nm) of the MLCT absorption band at 492 nm was observed. This kind of spectral change of compound **4** upon addition of Fe(II) ions indicated that compound **4** formed a complex with Fe(II) ions to form linear polyRuFe with Ru(II) and Fe(II) ions in alternate positions. Further increase of the amount of Fe(II) ions in the complexed mixture did not show a significant change in the intensity of the MLCT band at 577 nm, suggesting that polyRuFe is stable in solution.

Finally, polyRuFe was synthesized by refluxing compound **4** and Fe(BF_4_)_2_ 6H_2_O (1:1 molar ratio) in CHCl_3_/MeOH (1:1, *v*/*v*) for 24 h (Scheme 1). The polymer was isolated as a deep brown precipitate which was filtered, repeatedly washed with MeOH and CHCl_3_, and finally dried under vacuum overnight. PolyRuFe was obtained with 85% yield (see Materials and Method section for details of the synthetic procedure and Supplementary Materials for the NMR spectrum of the polymer). The solubility of polyRuFe was tested in various solvents. It was observed that the polymer is mainly soluble in highly boiling DMSO and DMF. The ^1^H NMR spectrum of polyRuFe displayed broad proton signals compared with that of compound **4**, confirming the polymer formation (Figure S7). The molecular weight (*M*_w_) of polyRuFe was estimated using the right-angle light scattering (RALLS) method and DMSO as a solvent. The molecular weight of the polymer was calculated to be 1.6 × 10^6^ Da. Such a high molecular weight is generally observed for MSPs and is sometime due to the formation of molecular aggregates in solution [13]. The thermal stability of polyRuFe was determined by thermogravimetric (TGA) analysis under an N_2_ atmosphere. TGA analysis revealed two degradation points; one corresponded to the breaking of the polymer chain at around 420 °C, and the other to the breaking of the ligand backbone at around 630 °C, suggesting high thermal stability of polyRuFe (Figure S8).

**Figure 3:**
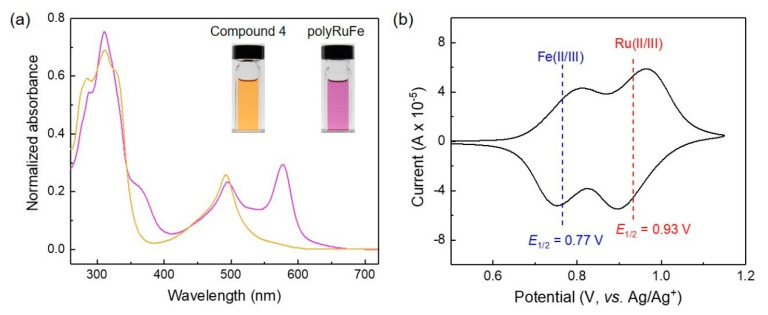
(**a**) UV–vis spectra of compound **4** (5 × 10^−6^ M in DCM/MeOH 1:1, *v*/*v*) and polyRuFe (5 × 10^−6^ M in DMSO), with their color in solution (inset). (**b**) Cyclic voltammogram of polyRuFe in a three-electrode system (glassy carbon as a working electrode, platinum flag as a counter electrode, and Ag/Ag^+^ as a reference electrode; electrolyte: 0.1 M LiClO_4_ in CH_3_CN, scan rate 50 mV/s).

The optical properties of polyRuFe (5 × 10^−6^ M in DMSO) were investigated by UV–vis absorption spectroscopy; the spectrum is shown in Figure 3a. For comparison, the absorption spectra of compound **4** (5 × 10^−6^ M in DCM/MeOH 1:1, *v*/*v*) and polyRuFe are displayed too. As mentioned earlier, compound **4** showed only two absorption bands at 311 and 495 nm, corresponding to the π–π* transition and MLCT absorption of the Ru(II)–terpyridine complex, respectively. However, the absorption spectrum of polyRuFe exhibited a broad absorption window including the π–π* transition at 311 nm, MLCT absorption of the Ru(II)–terpyridine complex portion at 492 nm, and MLCT absorption of the Fe(II)–terpyridine complex portion at 577 nm. Such a broad optical window originated from the coupling of two heterometallic complexes of Ru(II) and Fe(II) ions, clearly indicating the formation of polyRuFe. The formation of polyRuFe was further confirmed by the optical color change of the solution when transitioning from compound **4** to polyRuFe. Compound **4** exhibited a deep yellow color in solution due to the presence of the MLCT transition for the Ru(II)–terpyridine complex only, whereas polyRuFe exhibited a pink color in solution, due to the presence of MLCT transitions for both Ru(II)–terpyridine and Fe(II)–terpyridine complexes (Figure 3a: inset). 

We also investigated the electrochemical properties of polyRuFe, as it contains the redox-active metal ions Ru(II) and Fe(II). The electrochemical properties were investigated by cyclic voltammetry (CV) in a three.electrode system (a glassy carbon electrode containing a drop-casted polymer as the working electrode, a platinum flag as the counter electrode, Ag/Ag^+^ as the reference electrode; 0.1 M LiClO_4_ in CH_3_CN was used as the electrolyte). The CV spectrum of polyRuFe exhibited two distinct reversible one-electron redox processes due to the Fe(II)/Fe(III) and Ru(II)/Ru(III) redox pairs, further suggesting the presence of two heterometal ions into the polymer (Figure 3b). The first reversible redox signal was for the Fe(II)/Fe(III) pair, and the second one was for the the Ru(II)/Ru(III) pair. Upon application of the positive forward bias on the polymer, a first oxidation of Fe(II) occurred, with a peak potential of 0.80 V; then, oxidation of Ru(II) occurred, with a peak potential of 0.96 V. In the positive backward bias, stepwise reduction of Ru(III) and Fe(III) occurred, with peak potentials of 0.90 V and 0.75 V, respectively. The half-wave redox potentials (*E*_1/2_) were calculated to be 0.55 V and 0.72 V at a scan rate of 50 mV/s for Fe(II)/Fe(III) and Ru(II)/Ru(III) pairs, respectively. To confirm the formation of polyRuFe from compound **4**, CV analysis of compound **4** was also performed; the spectrum is shown in Figure S9. Compound **4** showed only one-electron redox processes of the Ru(II)/Ru(III) pair in contrast to polyRuFe which showed two distinct redox peaks for the Fe(II)/Fe(III) and Ru(II)/Ru(III) redox pairs. The weak redox nature around 0.42 V of compound **4** could be assigned to the redox nature of free tpy units. The above-observed broad optical and electrochemical window of polyRuFe indicates its potential for various applications.

## 3. Materials and Methods 

All chemicals and solvents for material synthesis and characterization were purchased from commercial suppliers (Sigma-Aldrich, Wako, TCI and Kanto Chemical Co., Tokyo, Japan) and used as received. Neutral alumina (Al_2_O_3_) from Kanto Chemical Co. Inc. was used for column chromatography. For synthesis and spectroscopic measurements, anhydrous solvents and spectrophotometric-grade solvents were used, respectively. Distilled water from a Milli-Q purification system was used for the synthesis and the experiments, when required.

The NMR spectra were recorded on a JEOL-ECZ 400 MHz nuclear magnetic resonance instrument (JEOL Ltd., Tokyo, Japan). Chemical shifts are shown relative to tetramethylsilane (TMS) and given in parts per million (ppm). MALDI–TOF mass spectra were measured on an AXIMA-CFR, Shimadzu/Kratos TOF mass spectrometer (Shimadzu Corporation, Kyoto, Japan) using 1,8,9-trihydroxyanthracene as matrix. The molecular weight of polyRuFe was determined by RALLS on a Viscotek 270 Dual Detector (Malvern Panalytical, Malvern, UK) using DMSO as the solvent (flow rate: 0.50 mL/min). TGA was performed by using an SII TG/DTA 6200 instrument (Hitachi High-Tech Corporation, Tokyo, Japan) in a N_2_ environment with a 10 °C/min heating rate. The UV–vis spectra were recorded using a Shimadzu UV-2550 UV–visible spectrophotometer (Shimadzu Corporation, Kyoto, Japan). CV was measured using an ALS/CHI electrochemical workstation (CH Instruments, Inc., Austin, Texas, USA). A conventional three-electrode system (glassy carbon electrode as the working electrode, platinum flag as the counter electrode, and Ag/Ag^+^ electrode in CH_3_CN with 0.1 M TBAP + 0.01 M AgNO_3_ as the reference electrode) was used for CV measurements. A small volume of polymer solution (in DMSO) was drop-casted on a glassy carbon electrode and dried at room temperature.

### 3.1. Synthesis of Compound ***2***


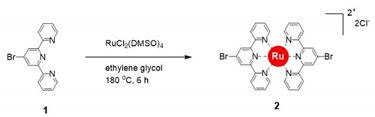


Compound **1** (4′-Bromo-2,2′:6′,2″-terpyridine, 686.75 mg, 2.2 mmol) and RuCl_2_(DMSO)_2_ (484.51 mg, 1.0 mmol) were placed in a 50 mL two-neck round-bottom flask under nitrogen atmosphere, followed by the addition of 15 mL of degassed ethylene glycol, and then the mixture was stirred at 180 °C for 6 h. Then, the reaction mixture was cooled to room temperature, and 200 mL THF was added to it; the mixture was stirred for 1 h. A precipitate formed, which was filtered off to obtain the crude product. The crude product was purified by column chromatography (neutral Al_2_O_3_), eluting with DCM/MeOH (4:1) to isolate compound **2** as a brown solid (724.64 mg, 91% yield). ^1^H NMR (CD_2_Cl_2_/CD_3_OD [1:1, *v*/*v*], 400 MHz, ppm): δ 9.19 (s, 4H), 8.69 (d, 4H), 8.01 (t, 4H), 7.44 (d, 4H), 7.30 (t, 4H). ^13^C NMR (CD_2_Cl_2_/CD_3_OD [1:1, *v*/*v*], 400 MHz, ppm): δ 157.72, 156.79, 152.96, 139.40, 132.72, 129.13, 128.04, 126.16. MALDI-MS (*m*/*z*): 725.82 [1−2Cl^−^]^+^ (calculated *m*/*z* = 725.41).

### 3.2. Synthesis of Compound ***4***


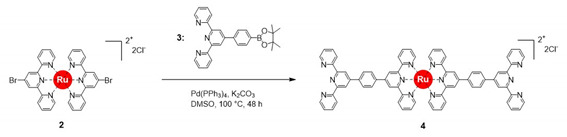


Compound **2** (183.15 mg, 0.23 mmol), compound **3** (500.62 mg, 1.15 mmol), Pd(PPh_3_)_4_ (40 mg, 0.034 mmol, 15%), and K_2_CO_3_ (80.2 mg, 0.58 mmol) were put in a 50 mL two-neck round-bottom flask under nitrogen atmosphere, followed by the addition of 20 mL anhydrous DMSO. The reaction mixture was heated at 100 °C for 48 h. DMSO was removed under reduced pressure, and the residue was purified by column chromatography (neutral Al_2_O_3_), eluting with DCM and DCM/MeOH (60:1, *v*/*v*) to isolate compound **4** as a red solid (219.06 mg, 76% yield). ^1^H NMR (CD_2_Cl_2_/CD_3_OD [1:1, *v*/*v*], 400 MHz, ppm) δ 9.30 (s, 4H), 8.90–8.88 (m, 8H), 8.77–8.75 (m, 8H), 8.49 (d, 4H), 8.37 (d, 4H). 8.07–8.03 (m, 8H), 7.54–7.51 (m, 8H), 7.33 (t, 4H). ^13^C NMR (DCM/MeOH [1:1, *v*/*v*], 400 MHz, ppm): δ 158.97, 157.03, 156.71, 156.40, 152.85, 149.97, 149.74, 149.12, 141.28, 139.26, 138.49, 138.01, 129.34, 129.22, 128.76, 125.83, 125.24, 122.73, 122.37, 119.54. MALDI-MS (*m*/*z*): 1182.78 [OsL1−2Cl^−^]^+^ (calculated *m*/*z* = 1182.33).

### 3.3. Synthesis of polyRuFe

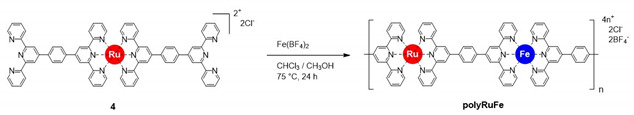



Compound **4** (40 mg, 0.03 mmol) was dissolved in 6 mL CHCl_3_ and 4 mL MeOH in a 25 mL round-bottom flask, followed by the addition of Fe(BF_4_)_2_ 6H_2_O (10.12 mg, 0.03 mmol) dissolved in 2 mL MeOH. The reaction mixture was heated at 75 °C for 24 h, then cooled to room temperature. A precipitate formed, which was filtered off and washed with CHCl_3_ and MeOH to get a deep brown residue of polyRuFe (42.6 mg, 85% yield). ^1^H NMR (DMSO-*d*_6_, 400 MHz, ppm) δ 9.96–9.78 (bd, 4H), 9.27–8.76 (bm, 8H), 8.18 (bs, 4H), 7.72–7.31 (bm, 8H). Elemental analysis calculated for [polyRuFe + 4CHCl_3_ + 6CH_3_OH] = C_82_H_76_N_12_O_6_FeRuB_2_Cl_14_F_8_: C, 45.89; H, 3.66; N, 7.82. Found: C, 46.09; H, 3.99; N, 7.32.

## 4. Conclusions

A heterobimetallic supramolecular polymer (polyRuFe) containing Ru(II) and Fe(II) was successfully synthesized by harnessing strongly and weak binding metal ions and was characterized by various spectroscopic techniques. A stepwise synthetic route was followed to obtain a high yield of product in each step as well as to introduce the heterometal ions in an alternate fashion into the polymer chain. The molecule 2,2′:6′,2″-terpyridine was used as a coordination ligand to introduce Ru(II) and Fe(II) in identical coordination environments. High thermal stability was observed for polyRuFe, with two degradation points: one around 420 °C corresponding to the breaking of the polymer chain, and the other at 630 °C corresponding to the breaking of the ligand backbone. Coupling of two heterometallic complexes into polyRuFe resulted in a wide absorption window starting from 311 nm to 577 nm. PolyRuFe was also associated with a broad electrochemical window with two distinct redox characteristics. Overall, this work described the synthesis of a new heterobimetallic supramolecular polymer (polyRuFe) containing homoleptic Ru(II) and Fe(II) complexes in an alternate way and showing broad optical and electrochemical windows. PolyRuFe can be of much interest for various optical, electrochemical, and electro-optical applications. For example, polyRuFe can be employed for voltage-tunable multicolor electrochromic applications. As polyRuFe contains two distinct redox waves with a broad optical window, we could selectively apply a voltage on the polymer film to oxidize the metal ions in a stepwise manner and get different types of electrochromism. More investigation in this direction is currently underway in our lab.

## Supplementary Materials

The following are available online. Figures S1–S7: NMR and MALDI mass spectra of compound **2**, compound **4,** and polyRuFe, Figure S8: TGA analysis of polyRuFe Figure S9: CV spectrum of compound **4**.

## Abbreviations

DCM	dichloromethane
MeOH	methanol
CHCl_3_	chloroform
DMSO	dimethyl sulfoxide
DMF	dimethyl formamide; CH_3_CN: acetonitrile
