# Supplementary material for: Synthesis of an Alternated Heterobimetallic Supramolecular Polymer Based on Ru(II) and Fe(II)
Source: Molecules. 2020 Nov 11;25(22):5261. doi: 10.3390/molecules25225261 (PMC7698060; doi:10.3390/molecules25225261)
Supplement: Supplementary file 1 [file molecules-25-05261-s001.pdf]

Supporting Information

Synthesis of Alternated Heterobimetallic Supramolecular Polymer Based on Ru(II) and Fe(II)

Manas Kumar Bera, Yoshikazu Ninomiya and Masayoshi Higuchi*

Electronic Functional Macromolecules Group, Research Center for Functional Materials, National Institute for Materials Science (NIMS), 1-1 Namiki, Tsukuba, Ibaraki 305-0044, Japan

Table of contents

Figure S1-S7. NMR and MALDI mass spectra of compound 2, compound 4 and polyRuFe.

Figure S8. TGA analysis of polyRuFe.

Figure S9. CV spectrum of compound 4.

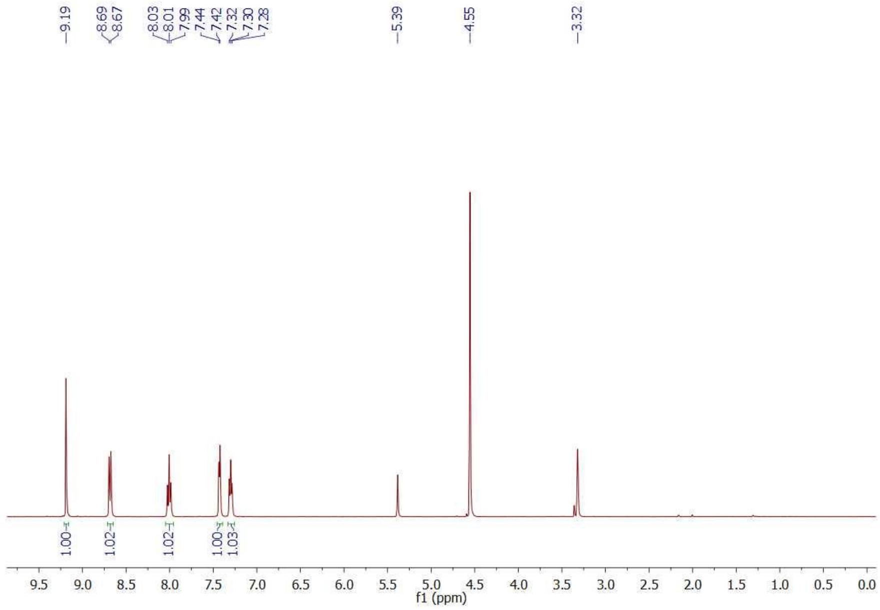

Figure S1. ^{1}H NMR spectrum of compound 2 in CD_2Cl_2/CD_3OD (1:1, v/v).

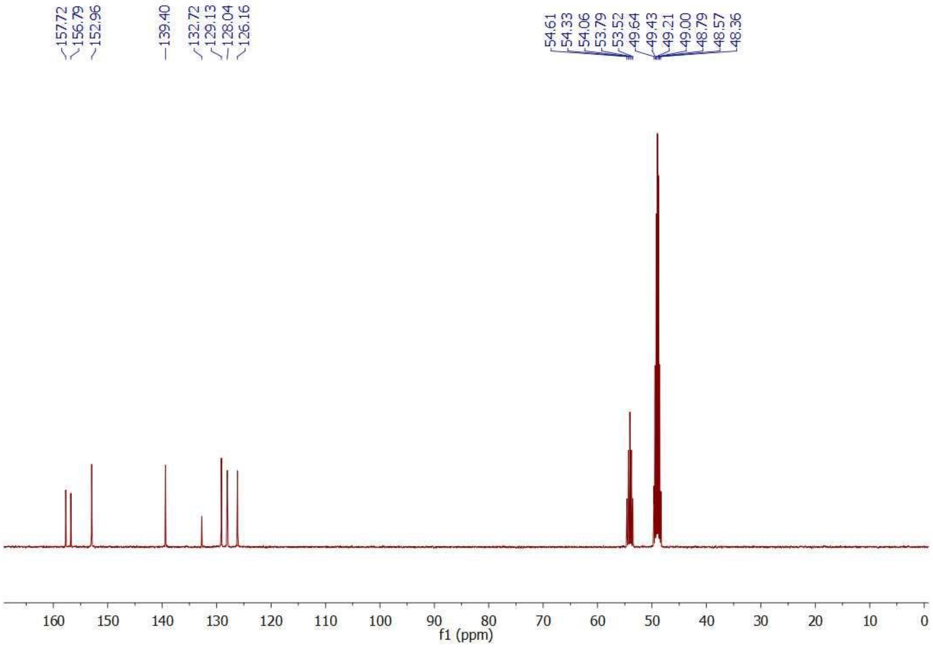

Figure S2. ^{13}C NMR spectrum of compound 2 in CD_2Cl_2/CD_3OD (1:1, v/v).

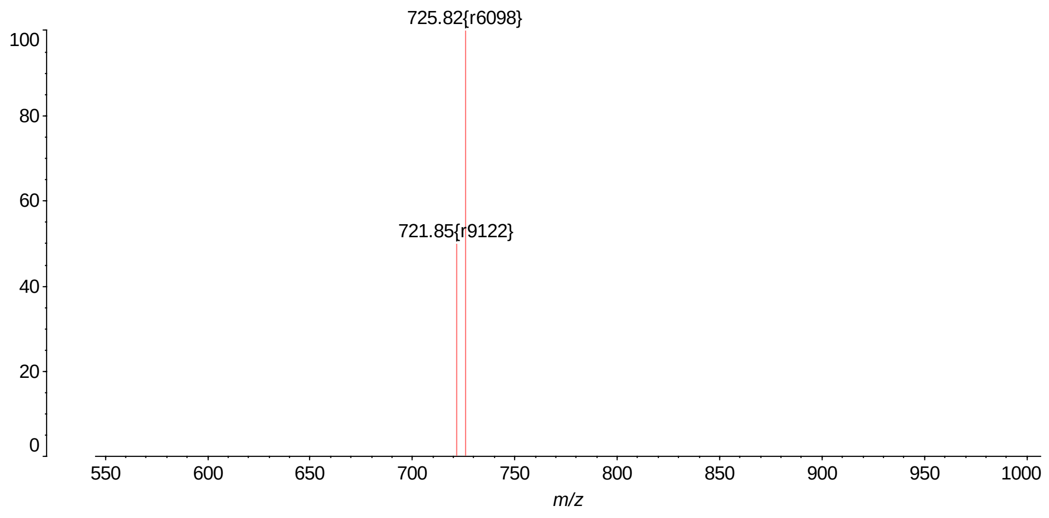

Figure S3. MALDI mass spectrum of compound 2.

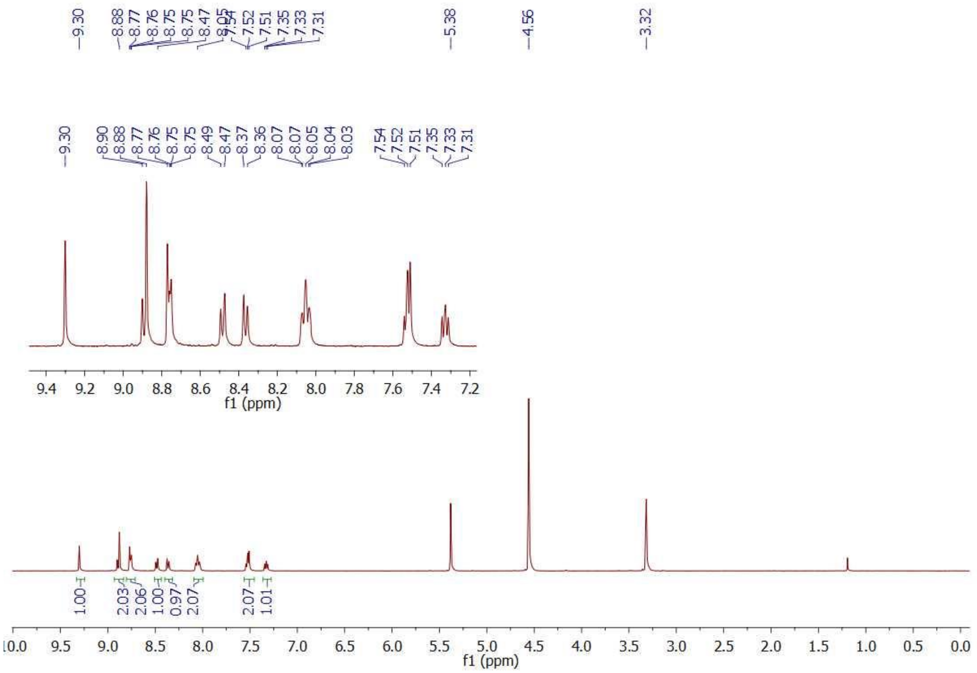

Figure S4. ^{1}H NMR spectrum of compound 4 in CD_2Cl_2/CD_3OD (1:1, v/v).

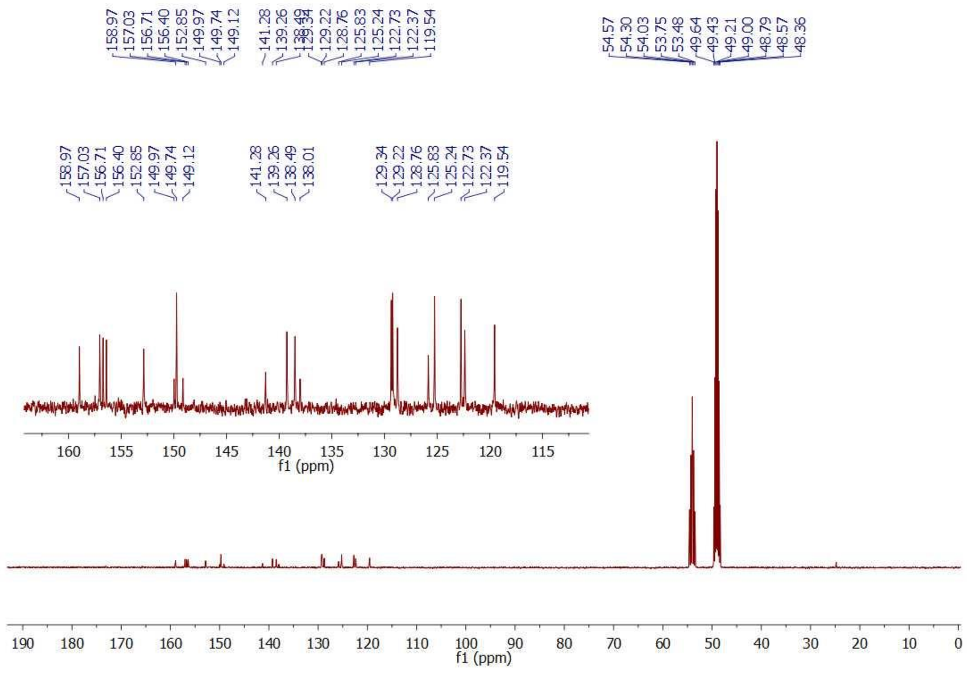

Figure S5. ^{13}C NMR spectrum of compound 4 in CD_2Cl_2/CD_3OD (1:1, v/v).

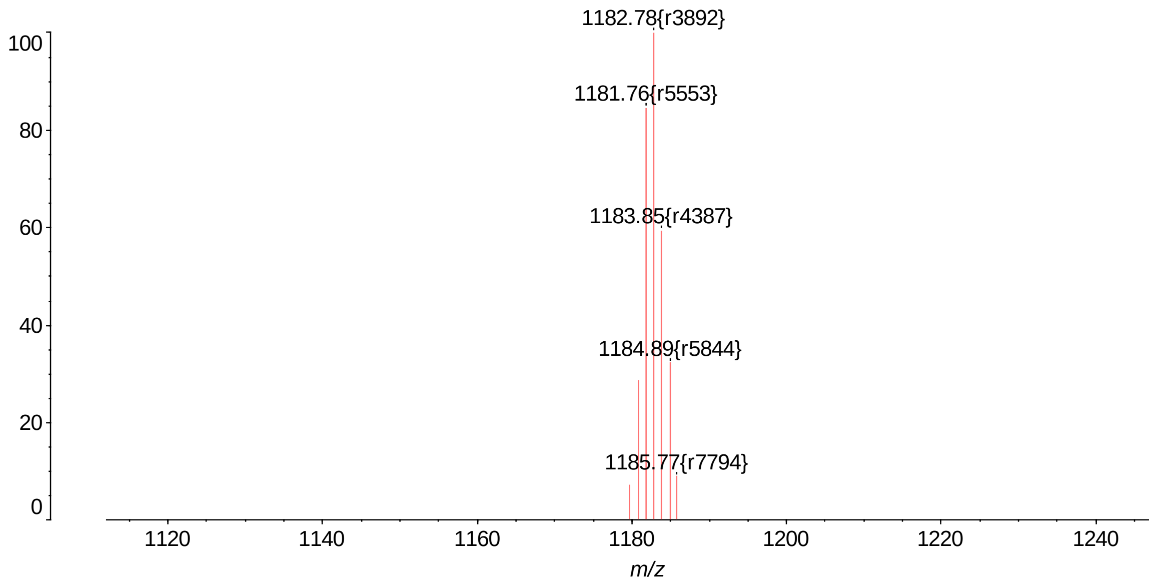

Figure S6. MALDI mass spectrum of compound 4.

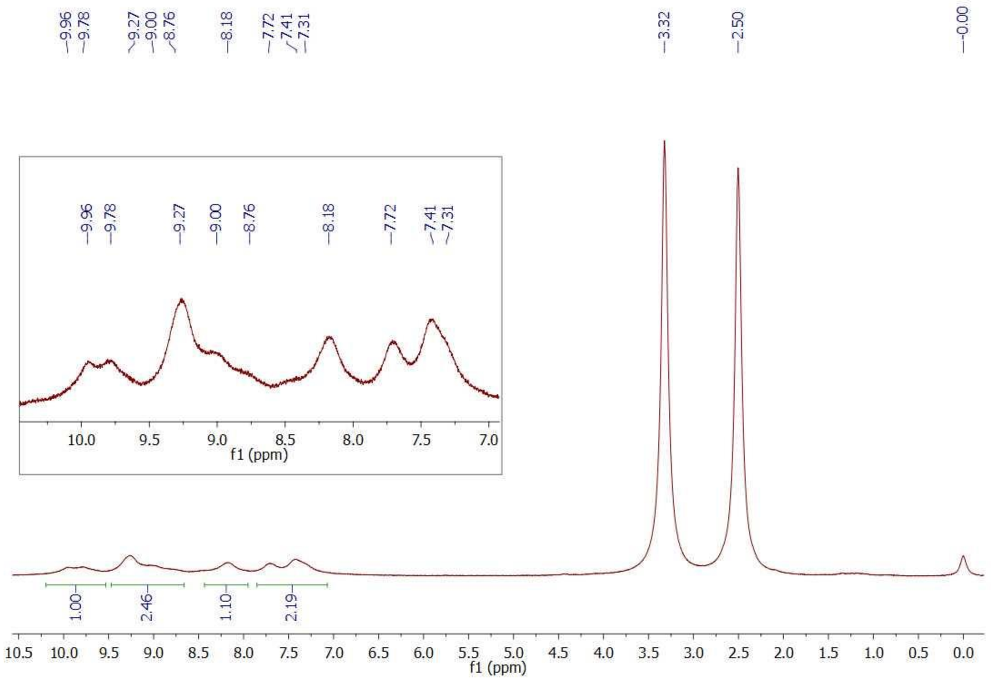

Figure S7. ^{1}H NMR spectrum of polyRuFe in DMSO-d_6.

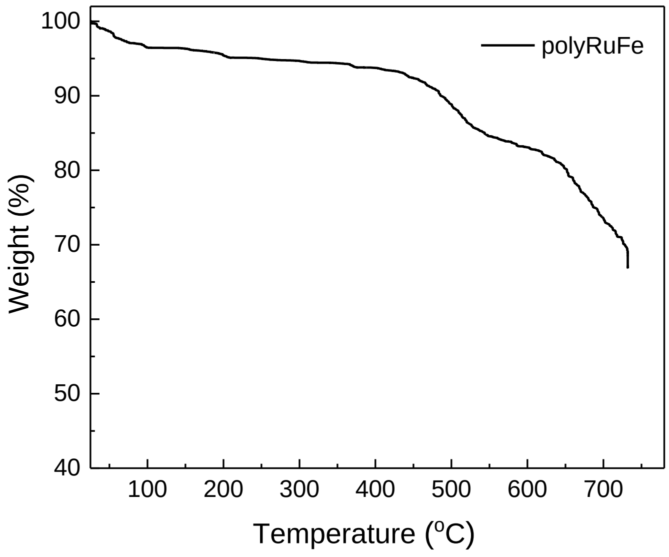

Figure S8. The TGA analysis of polyRuFe, showing high thermal stability with two degradation temperatures at around 420 and 630 °C, respectively.

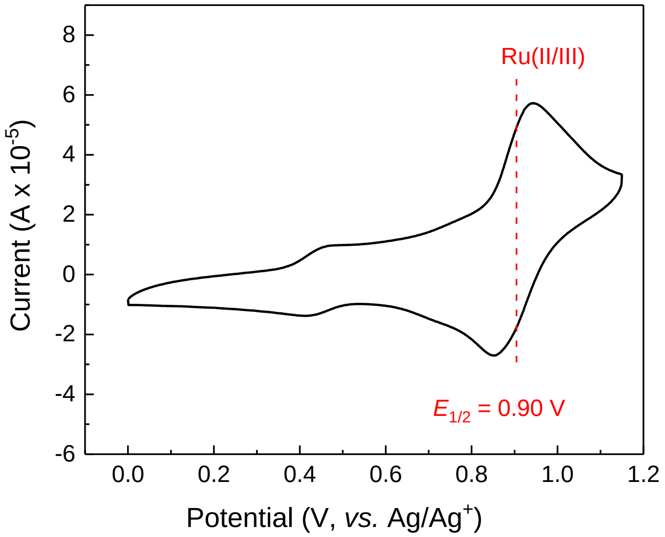

Figure S9. Cyclic voltammogram of compound 4 in three electrode system (glassy carbon as working electrode, platinum flag as counter electrode, and Ag/Ag^+ as reference electrode, electrolyte: 0.1 M $LiClO_4$ in CH_3CN, scan rate 50 mV/s).
